# Insights into Early Onset Dementia: a protocol for an 8-year nationwide retrospective study using administrative data

**DOI:** 10.1192/j.eurpsy.2024.1496

**Published:** 2024-08-27

**Authors:** B. F. Pinto, A. R. Ferreira, M. Gonçalves Pinho, A. Freitas, L. Fernandes

**Affiliations:** ^1^Faculty of Medicine, University of Porto; ^2^CINTESIS@RISE, Department of Clinical Neurosciences and Mental Health, Faculty of Medicina, University of Porto, Porto; ^3^Department of Psychiatry and Mental Health, Centro Hospitalar do Tâmega e Sousa, Penafiel; ^4^CINTESIS@RISE, Department of Community Medicine, Information and Health Decision Sciences (MEDCIDS), Faculty of Medicine, University of Porto; ^5^Psychiatry Service, Centro Hospitalar Universitário de São João, Porto, Portugal

## Abstract

**Introduction:**

Early-onset dementia (EOD) is defined as any type of dementia with an onset before the age of 65. Despite its profound impact on patients and their families, EOD has garnered less attention when compared to late-onset dementia (LOD), often resulting in its underestimation. In comparison to LOD, EOD commonly manifests with atypical and heterogeneous symptoms, encompassing mainly non-memory problems, ranging from language and executive impairments to behavioral-led dysfunction. Despite the importance of accurate data to organize appropriate healthcare, evidence regarding EOD patients in Portugal is lacking.

**Objectives:**

The primary aims of this study include identifying the causes for hospitalization in EOD patients, diagnosed with dementia either as a primary or secondary diagnosis, and comparing them with inpatients aged 65 and older (LOD). Additionally, the study aims to analyze key hospitalization outcomes for both groups, including length of stay, in-hospital mortality, and readmissions. As a secondary aim, this study seeks to describe subtypes of EOD.

**Methods:**

A retrospective observational study will be conducted following the RECORD statement. Data will be retrieved from an administrative database that gathers de-identified routinely collected hospitalization data from all Portuguese mainland public hospitals. Hospitalization episodes of inpatients younger than 65 years old, with a primary or secondary diagnosis of dementia (ascertained by ICD-9-CM codes 290.0-290.4, 294.0-294.2, 331.0, 331.1, and 331.82), will be extracted. Comparison patients will be selected by propensity score-matching from inpatients over 65 years with a dementia ICD-9-CM code (in any position), matched for Charlson Comorbidity Index (CCI).

**Results:**

Descriptive and analytical statistics will be conducted to describe and characterize both group of inpatients. Variables such as age at admission, sex, place of residence, causes and type of admission, psychiatric comorbidities, length of stay (LoS), destination after discharge, readmissions, in-hospital mortality and hospital charges will be analyzed.

**Conclusions:**

With this nationwide analysis of EOD hospitalizations, we aim to reveal critical aspects of this condition, including common causes of admission, diagnostic features and health outcomes, allowing for appropriate medical interventions and support tailored to the specific needs of this clinical group.

**Disclosure of Interest:**

None Declared

